# Clinician Willingness to Prescribe Medications for Opioid Use Disorder to Adolescents in Indiana

**DOI:** 10.1001/jamanetworkopen.2024.35416

**Published:** 2024-09-25

**Authors:** Matthew C. Aalsma, Lauren A. Bell, Katherine Schwartz, Fangqian Ouyang, Marynia Kolak, Patrick O. Monahan, Sharon P. Mermelstein, Ian Carson, Leslie A. Hulvershorn, Zachary W. Adams

**Affiliations:** 1 Department of Pediatrics, Indiana University School of Medicine, Indianapolis; 2 Department of Preventive Medicine, University of Tennessee Health Science Center, Memphis; 3 Department of Biostatistics and Health Data Science, Indiana University School of Medicine, Indianapolis; 4 Department of Geography and Geographic Information Science, School of Earth, Society, and Environment, University of Illinois Urbana-Champaign, Urbana; 5 Department of Biostatistics and Health Data Science, School of Medicine, Fairbanks School of Public Health, Simon Comprehensive Cancer Center, Indiana University, Indianapolis; 6 Department of Psychiatry, Indiana University School of Medicine, Indianapolis; 7 Department of Psychology, School of Science, Indiana University Indianapolis, Indianapolis

## Abstract

**Importance:**

Prescribing medications for opioid use disorders (MOUD), including buprenorphine, naltrexone, and methadone, to adolescents remains an underused evidence-based strategy for reducing harms associated with opioid use.

**Objective:**

To identify potential associations between clinician- and community-level characteristics regarding clinicians’ self-reported willingness to prescribe MOUD to adolescents.

**Design, Setting, and Participants:**

This cross-sectional study included a phone survey of Indiana clinicians and spatial analysis of community-level characteristics. Clinicians were eligible for inclusion in analyses if actively providing health care and listed on the Buprenorphine Practitioner Locator website, a publicly available national registry of clinicians possessing a waiver to legally prescribe buprenorphine (ie, waivered clinicians).

**Exposures:**

Community-level characteristics, including total population, rurality or urbanicity, percentage with incomes below the federal poverty line, and racial or ethnic makeup.

**Main Outcomes and Measures:**

Clinicians were asked about their willingness to prescribe MOUD to adolescents younger than 18 years if clinically indicated. Responses were recorded as no, yes, or yes with conditions.

**Results:**

Among the 871 clinicians listed on the website as of July 2022, 832 were eligible for inclusion and contacted by phone. Among waivered clinicians, 759 (91.2%) reported being unwilling to prescribe MOUD to adolescents, 73 clinicians (8.8%) reported willingness to prescribe MOUD to adolescents, and only 24 (2.9%) would do so without conditions. A multivariable logistic regression model including spatially lagged community-level variables showed that, among areas with waivered clinicians, clinicians practicing in more populated areas were significantly less likely to prescribe to adolescents (β = 0.65; 95% CI, 0.49-0.87; *P* = .003). Similarly, those in more rural areas were significantly more likely to prescribe to adolescents (β = 1.27; 95% CI, 1.02-1.58; *P* = .03). Variation in clinician willingness to prescribe was not explained by other community-level characteristics. Among all waivered clinicians, advanced practice clinicians were less likely than physicians to report willingness to prescribe (β = 0.58; 95% CI, 0.35-0.97; *P* = .04), as were physicians without any specialty training relevant to MOUD prescribing when compared with family medicine clinicians (β = 0.40; 95% CI, 0.18-0.89; *P* = .03). A small subgroup of waivered clinicians had training in pediatrics (13 clinicians [1.6%]), and none were willing to prescribe MOUD to adolescents.

**Conclusions and Relevance:**

From this cross-sectional study, it appears that Indiana adolescents continued to face gaps in access to MOUD treatment, despite its well-established efficacy. Programs that support primary care practitioners, including family medicine clinicians and pediatricians, in safe and appropriate use of MOUD in adolescents may bridge these gaps.

## Key Points

**Question:**

Are clinician- and community-level characteristics associated with willingness to prescribe medications for opioid use disorder (MOUD) to adolescents among clinicians possessing a waiver to legally prescribe buprenorphine (ie, waivered clinicians)?

**Findings:**

In this cross-sectional study of 832 waivered clinicians in Indiana, 91.2% reported unwillingness to prescribe MOUD to adolescents. Clinicians trained in family medicine and those serving in less populated communities were significantly more likely than others to prescribe to adolescents.

**Meaning:**

These findings suggest that the average parent in Indiana would need to call 11 waivered clinicians to find 1 willing to prescribe MOUD to adolescents.

## Teaser

This cross-sectional study identifies potential associations between clinician and community characteristics regarding clinicians’ self-reported willingness to prescribe medications for opioid use disorder to adolescents.

## Introduction

Increased access to opioids and the ubiquity of fentanyl in counterfeit pills and other common drugs^1^ have put adolescents at particular risk of overdose, given that even experimental drug use can result in death.^2,3^ Yet, treating opioid use disorders (OUD) with FDA-approved medications (ie, medications for opioid use disorder [MOUD]; buprenorphine, naltrexone, methadone) remains an underused evidence-based strategy for reducing harms associated with opioid use and preventing overdose, especially among adolescents.^4^

Buprenorphine is the only FDA-approved MOUD for treating individuals as young as 16 years, though other MOUD (naltrexone, methadone) are prescribed off-label to youth younger than 18 years. Randomized clinical trials have demonstrated buprenorphine is both safe and effective at promoting opioid abstinence, treatment engagement, and reduced risk of overdose death.^5,6,7,8^ Use of MOUD to treat adolescents with OUD has been recommended by the American Academy of Pediatrics,^9^ the Society for Adolescent Medicine,^10^ and the American Academy of Child and Adolescent Psychiatry.^11^ Despite this widespread endorsement, adolescents rarely use MOUD.^12^ In a recent study^13^ of Ohio adolescents enrolled in Medicaid, only 4% of enrollees with OUD received MOUD. National Medicaid data from 2015 to 2020 indicated, while buprenorphine prescribing for adults increased by 47%, prescribing for adolescents decreased by 45%.^14^ Among youth ages 12 to 17 years who reported recent daily opioid use and/or a history of injection drug use in a national survey, 13% received MOUD.^15^ A 2022 secret shopper study^4^ found that, among 160 residential addiction treatment facilities admitting adolescents, fewer than 25% would initiate buprenorphine with patients aged 16 years.

Clinicians commonly report barriers to prescribing MOUD to adolescents. In a study of addiction treatment clinicians in Georgia, concerns included MOUD prescription misuse or diversion and the effects of MOUD on adolescents’ developing brains, including potential for long-term dependence.^16^ Additional clinician-reported barriers to prescribing MOUD have included limited capacity to treat an anticipated influx of patients, stigma toward individuals who use drugs, lack of mental health cotreatment access, and low MOUD competence.^17,18,19,20^ Until January 2024, clinicians faced the X-waiver barrier, which required buprenorphine prescribers to complete training, obtain federal certification, and limit the number of patients treated.^21^

Compounded with clinician-related barriers to prescribing MOUD to adolescents, young people face community-level barriers to treatment. Minoritized adults with OUD, including Black and Hispanic or Latine individuals and those living in lower income areas, are less likely to access evidence-based OUD treatment, including MOUD.^12,22^ Residents of rural communities experience longer travel times and limited access to health services, including MOUD.^23,24^ Given these barriers, our research team aimed to assess the availability of MOUD for adolescents in Indiana and explore associations between both clinician- and community-level characteristics on clinician willingness to prescribe MOUD to adolescents.

## Methods

This cross-sectional study was approved by the institutional review board at Indiana University, and verbal informed consent was waived due to the low risk of the study. This study followed the Strengthening the Reporting of Observational Studies in Epidemiology (STROBE) reporting guideline.

### Data Collection

All data collection occurred in July and August 2022 with the purpose of facilitating a statewide service, Indiana’s Adolescent Addiction Access (AAA) program. Clinician-level data reported herein were not collected for research purposes. Since May 2021, AAA has provided statewide phone consultation for adolescent health care clinicians (eg, primary care clinicians, emergency department (ED) physicians) seeking up-to-date information regarding adolescent substance use or to locate other clinicians willing to care for adolescents who use drugs. In response to calls by the Substance Abuse and Mental Health Administration (SAMHSA) to make buprenorphine available to adolescents experiencing OUD, AAA staff sought to expand their existing lists of resources to include contact information for local clinicians most likely to prescribe MOUD (ie, buprenorphine, naltrexone, methadone) to adolescents. To do so, AAA staff first accessed SAMHSA’s publicly available list of waivered clinicians, the Buprenorphine Practitioner Locator^25^ to collect clinician names, addresses, phone numbers, and credentials. At the time of data collection, inclusion on the SAMHSA list of waivered clinicians indicated an individual’s legal ability to prescribe buprenorphine under the so-called X-Waiver and voluntary registration on the SAMHSA site. For AAA’s purposes, site registration served as evidence of clinicians’ affirmative endorsement of MOUD as a viable treatment option. As of 2023, all practitioners with the Drug Enforcement Administration (DEA) Schedules II-V on their DEA registration are legally able to prescribe buprenorphine without obtaining a waiver.^25^

AAA staff attempted to contact all 871 waivered clinicians listed on SAMHSA’s site in Indiana and reached 832 clinicians (95.5%).^25^ Clinicians were phoned repeatedly using the number listed on the website, with staff leaving messages until a response or return call was attained. AAA staff spoke directly to the clinician listed or their direct-report nurse, asking, “Would you provide MOUD services for anyone younger than 18-years-old if clinically indicated?” If the response was no, AAA staff asked the same question regarding adolescents aged 16 or 17 years. If the response was yes, then the clinician’s name was added to the AAA list of those willing to prescribe MOUD to adolescents. Clinician credentials (eg, MD vs advanced practice clinician [APC; ie, nurse practitioner or physician’s assistant]) and specialty training and/or board certification (eg, emergency medicine, psychiatry) were recorded or confirmed based on searches of Doximity.

The research team accessed community-level contextual data from the Opioid Environment Policy Scan Data Warehouse.^26^ These data describe the sociodemographic characteristics of all of Indiana’s zip code tabulation areas (ZCTAs),^27^ including the 179 ZCTAs in which Indiana’s waivered clinicians were located. Sociodemographic characteristics are 5-year estimates from 2018 and originally sourced from the American Community Survey.^26^

### Statistical Analysis

We descriptively reported waivered clinician willingness to prescribe to adolescents (no, do not prescribe; yes, prescribe; yes, prescribe with conditions) by their credentialing and specialty training. Clinician-level data were linked with Indiana’s community-level data by unique ZCTA. For ZCTAs in which more than 1 waivered clinician was located, ZCTAs were categorized as including a clinician willing to prescribe MOUD to adolescents if at least 1 clinician within the ZCTA was willing to prescribe. Logistic regression models were used to investigate associations between community characteristics and clinician willingness to prescribe to adolescents, while using generalized estimating equations (GEE) to account for clustering of clinicians within ZCTAs. At the community level (ie, ZCTAs), we used analysis of variance (ANOVA) F test to compare 3 clinician groups (no waivered clinician; waivered clinicians, will not prescribe; waivered clinicians, will prescribe) on community-level variables of interest included total population, percentage of population identified as Black or African American, percentage of the population earning below the federal poverty rate, and a measure of rurality or urbanicity, which was described with Rural Urban Commuting Area version 2 codes (RUCA2), an index from 1 to 10, with higher scores indicating rurality.^28^ All tests were based on a 2-sided significance level of *P* < .05.

Logistic regression models do not account for wider distribution of sociodemographic characteristics in areas surrounding the single ZCTAs in which waivered clinicians are located. Therefore, we calculated Global Moran *I* for variables of interest to test for spatial dependency. A spatial weight matrix encoding neighborhood associations for all ZCTAs in Indiana, not just clinician-level areal units, was calculated. We used a first-order queen contiguity as the spatial weight matrix, meaning that neighbors share a boundary. Moran *I* test confirmed a highly significant spatial autocorrelation, indicating that observations are not independent.

To estimate spatial spillover effects of nearby areas on ZCTAs in which waivered clinicians are located, we calculated spatial lags of variables hypothesized to influence clinician decision-making. A spatial lag interacts the variable of interest and the average of nearby areas using the spatial weight matrix calculated. First, we conducted a bivariable logistic regression on these spatially lagged variables. Then, we calculated a multivariable logistic regression with clinician characteristics, sociodemographic characteristics of clinician locations, and selected spatially lagged explanatory variables, or SLX model, as y = Xβ + WXθ + ε. In this model, while β contains the parameters of the direct effect estimates, W is the spatial weights matrix, and θ contains the parameters of the spatial spillover effects averaged among the immediate location’s neighbors. SLX models are simplified spatial econometric regressions that do not require specialized estimation methods and may retain unbiased estimators of θ, allowing us to examine whether a change in the value of the local estimators, as well as those in the neighboring areas, are associated with local clinicians’ willingness to prescribe MOUD to adolescents. We compared performance of the SLX model to the standard nonspatial multivariable logistic regression model. We calculated spatial weight matrix, Moran *I*, and spatially lagged estimators in Geoda; models were estimated using SAS version 9.4 (SAS Institute).

## Results

From the 871 individual waivered buprenorphine clinicians listed on the SAMHSA website, we culled duplicate listings or clinicians who were no longer practicing, resulting in a final list of 832 practitioners eligible for inclusion in analyses. Only 24 (2.9%) clinicians reported willingness to prescribe to adolescents with no conditions, and 759 (91.2%) stated they would not prescribe MOUD to adolescents. An additional 49 (5.9%) clinicians stated they would prescribe to adolescents under specific conditions, including if the adolescent was at least 16 years old (10 [1.2%]), if the adolescent’s health insurance would cover MOUD (21 [2.5%]), or under other varied conditions (18 [2.2%]). A description of waivered clinicians by willingness to prescribe MOUD to adolescents can be found in Table 1. Waivered clinicians included a roughly equal mix of physicians (476 MDs and DOs [57.2%]) and APCs (356 [42.8%]. Waivered clinicians varied widely in their specialty training. More than two-thirds of clinicians had training in categories potentially relevant to prescribing MOUD to adolescents, namely training in behavioral health issues (addiction medicine or psychiatry) or in treating younger patients (family medicine or pediatrics). Among all waivered clinicians, advanced practice clinicians were less likely than physicians to report willingness to prescribe (β = 0.58; 95% CI, 0.35-0.97; *P* = .04), as were physicians without any specialty training relevant to MOUD prescribing when compared with family medicine clinicians (β = 0.40; 95% CI, 0.18-0.89; *P* = .03). A small subgroup of waivered clinicians had training in pediatrics (13 clinicians [1.6%]), and none were willing to prescribe MOUD to adolescents.

**Table 1.** Characteristics of Indiana’s Waivered Clinicians by Willingness to Prescribe MOUD to Adolescents

Characteristics	Total, No. (%)	Clinician willingness to prescribe MOUD to adolescents, No. (%)		
		No, do not prescribe (n = 759)	Yes, prescribe (n = 24)	Yes, prescribe with conditions (n = 49)
Clinician credentials, Total No.	832	759	24	49
MD or DO	476 (57.2)	429 (90.1)	16 (3.4)	31 (6.5)
APC^a^	356 (42.8)	330 (92.7)	8 (2.2)	18 (5.1)
Clinician training^b^				
Addiction medicine	55 (6.6)	46 (83.6)	4 (7.3)	5 (9.1)
Psychiatry	202 (24.3)	185 (91.6)	12 (5.9)	5 (2.5)
Family medicine	308 (37.0)	270 (87.7)	7 (2.3)	31 (10.0)
Pediatrics	13 (1.6)	13 (100.0)	0	0
Other^c^	202 (24.3)	194 (96.0)	1 (0.5)	7 (3.5)
Unknown	52 (6.3)	51 (98.1)	0	1 (1.9)

Abbreviations: APC, advanced practice clinician; DO, doctor of osteopathic medicine; MD, medical doctor; MOUD, medications for opioid use disorder.

^a^ Advanced practice clinician (ie, nurse practitioner, physician’s assistant).

^b^ Though clinicians could have completed training in more than 1 specialty, clinicians were counted in the first category in which they had training as rank ordered here (eg, clinicians counted as having training in family medicine did not have training in addiction medicine or psychiatry).

^c^ Clinicians included in the other training category did not have specialty training associated with behavioral health issues (ie, addiction medicine or psychiatry) or training associated with caring for adolescents (ie, family medicine or pediatrics) and instead had a variety of unrelated specialty training (eg, geriatrics, sleep medicine, surgery, internal medicine).

**Figure 1.**
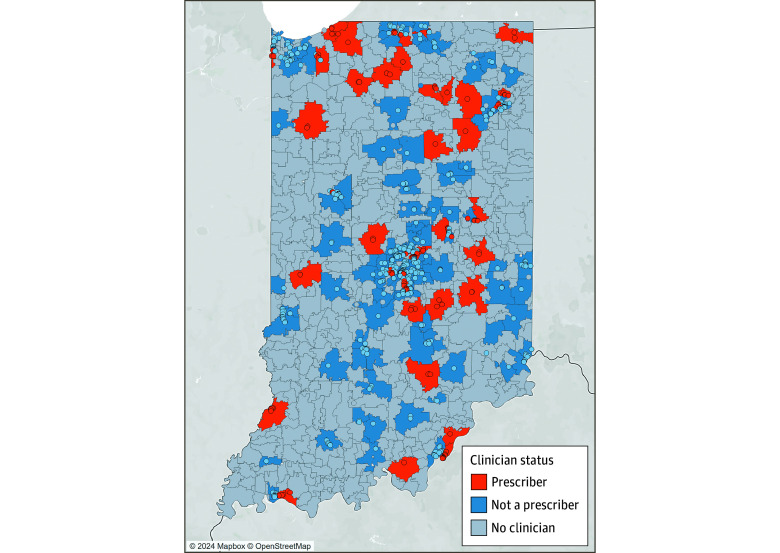
Locations of Waivered Clinicians and Willingness to Prescribe Medications for Opioid Use Disorder to Adolescents Within Indiana’s Zip Code Tabulation Areas

**Figure 2.**
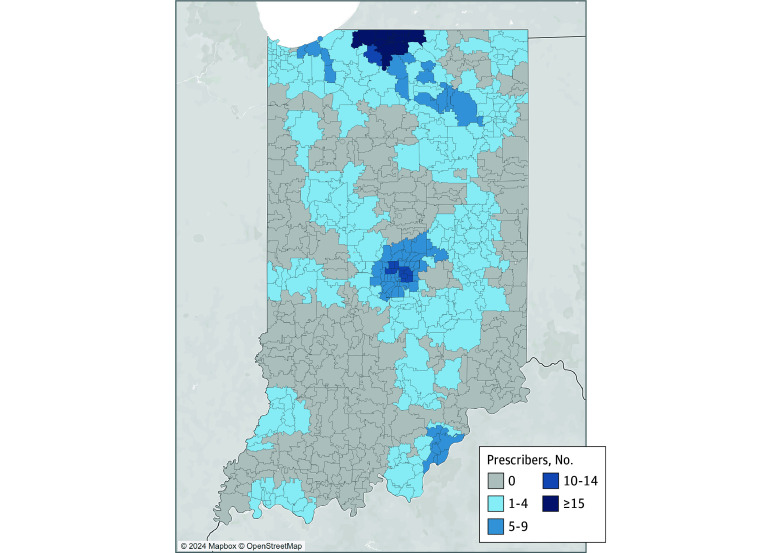
Locations of Waivered Clinicians Willing to Prescribe Medications for Opioid Use Disorder to Adolescents Within 30 Minutes of Zip Code Tabulation Area Centroid

Figure 1 and Figure 2 depict the statewide distribution of waivered buprenorphine clinicians and their willingness to provide MOUD to adolescents. Table 2 describes the community-level characteristics of all Indiana ZCTAs by the presence or absence of a waivered clinician and by clinician willingness to prescribe MOUDs to adolescents. ANOVAs indicated significant differences in characteristics by clinician prescribing status. As depicted in Figure 1, there were no waivered clinicians in most of Indiana’s 807 ZCTAs (628 [77.8%]). When compared with areas with waivered clinicians, locations without waivered clinicians tended to be less populated, have a smaller proportion of Black or African American residents and individuals living with incomes below the federal poverty line, and considered more rural. Among areas where waivered clinicians practice, practice areas with at least 1 clinician willing to prescribe MOUDs to adolescents were more likely to be considered rural and had a smaller proportion of Black or African American residents and individuals living with incomes below the federal poverty line than practice areas including only waivered clinicians unwilling to prescribe to adolescents.

**Table 2.** Description of Indiana’s ZCTAs by Presence or Absence of Waivered Clinician and Willingness to Prescribe MOUD to Adolescents

Characteristics	Mean (SD)				ANOVA *P* value (comparison of 3 ZCTA groups)
	Populated ZCTAs in Indiana (n = 807)	No waivered clinician (n = 628)	Clinician willingness to prescribe MOUD to adolescents		
			No, will not prescribe (n = 136)	Yes, will prescribe (n = 43)	
Total population (in 10 000)	0.9 (1.2)	0.4 (0.6)	2.3 (1.3)	2.3 (1.3)	<.001
% Population Black or African American	3.9 (11.0)	1.9 (7.4)	12.0 (18.5)	7.5 (10.2)	<.001
% Population with incomes below the federal poverty line	12.9 (9.9)	12.3 (10.0)	15.2 (9.3)	14.5 (9.2)	.005
Rurality^a^	3.7 (3.0)	4.2 (3.0)	2.1 (2.3)	2.8 (2.3)	<.001

Abbreviations: ANOVA, analysis of variance; MOUD, medications for opioid use disorder; ZCTA, zip code tabulation areas.

^a^ Rurality is described with Rural Urban Commuting Area, version 2 (RUCA2) codes, an index from 1-10 in which higher scores indicate a location is more rural.^29^

Table 3 describes 2 multivariable regression models estimating clinician willingness to prescribe MOUD to adolescents (yes or no). The standard logistic regression model includes all clinician- and community-level variables of interest, and accounts for clustering of clinicians within ZCTA. The SLX model incorporates the spatially lagged sociodemographic variables (Table 3), accounting for potential spillover effects of neighboring locations on clinician willingness to prescribe to adolescents. Both models revealed significant associations between characteristics of the clinicians’ location and their willingness to prescribe; waivered clinicians located in less populated areas (β = 0.65; 95% CI, 0.49-0.87; *P* = .003) and more rural areas were more likely to prescribe to adolescents (β = 1.27; 95% CI, 1.02-1.58; *P* = .03). No significant associations were found between clinician willingness to prescribe and sociodemographic characteristics of the residents living within the clinician’s ZCTA. Results of both models indicated no spillover effects of the characteristics of areas neighboring clinicians’ local ZCTAs. In contrast, QIC statistics indicated better model fit for the SLX model (QIC, 450) than the standard model without spatially lagged variables (QIC, 470).

**Table 3.** Multivariable Logistic Regression Models With Dependent Variable of Clinician Willingness to Prescribe MOUD to Adolescents^a^

Variable	Standard model		SLX model	
	Estimate	Probability χ^2^	Estimate	Probability χ^2^
Sociodemographic characteristics of clinician location and neighboring locations				
Total population (every 10 000 people increase)	0.68 (0.50-0.91)	0.01	0.65 (0.49-0.87)	0.004
Total population (W)^b^	NA	NA	0.79 (0.47-1.35)	0.40
% Black or African American (every percentage increase)	0.99 (0.97-1.01)	0.42	1.01 (0.97-1.06)	0.52
% Black or African American (W)	NA	NA	0.97 (0.92-1.03)	0.38
% With incomes below the federal poverty line (every percentage increase)	1.02 (0.97-1.07)	0.44	1.04 (0.98-1.09)	0.18
% With incomes below the federal poverty line (W)	NA	NA	0.92 (0.84-1.01)	0.09
Rurality (every 1 point increase [1-10])^c^	1.15 (0.99-1.33)	0.07	1.27 (1.02-1.58)	0.03
Rurality (W)	NA	NA	0.75 (0.54-1.04)	0.09
Clinician characteristics				
Clinician credentials				
MD/DO	1 [Reference]	NA	1 [Reference]	NA
APC	0.62 (0.38-0.99)	0.04	0.58 (0.35-0.97)	0.04
Clinician training				
Family medicine	1 [Reference]	NA	1 [Reference]	NA
Addiction medicine or psychiatry	1.07 (0.62-1.86)	0.81	1.13 (0.60-2.10)	0.71
Other^d^	0.41 (0.20-0.83)	0.01	0.40 (0.18-0.89)	0.03

Abbreviations: APC, advanced practice clinician (ie, nurse practitioner, physician’s assistant); DO, doctor of osteopathic medicine; MD, medical doctor; NA, not applicable.

^a^ Both the standard model (QIC statistic, 470) and the SLX model (QIC statistic, 450) account for clustering of clinicians within zip code tabulation areas.

^b^ W, spatially lagged variable, generated by spatial weight matrices.

^c^ Rurality is described with Rural Urban Commuting Area, version 2 (RUCA2) codes, an index from 1-10 in which higher scores indicate a location is more rural.^29^

^d^ Other, clinician had training in any category other than addiction medicine, psychiatry, or family medicine.

Both models also indicated significant associations between clinician characteristics and their willingness to prescribe MOUD to adolescents. In both models, physicians were more likely than APCs to prescribe MOUD. Waivered family medicine clinicians were more likely to prescribe than clinicians without any specialty training relevant to prescribing MOUD. Waivered family medicine clinicians’ odds of willingness to prescribe to adolescents were not significantly different from those of waivered addiction medicine-trained and psychiatry-trained or credentialed clinicians. Bivariable results are available in the eTable in Supplement 1. In the bivariable SLX model, community-level racial makeup was associated with willingness to prescribe, but this association was nonsignificant when other variables, such as community poverty rates were adjusted for in the multivariable models.

## Discussion

This exploration of Indiana’s waivered clinicians highlighted the continued absence of evidence-based treatment options available to youth with OUD and showed how few areas were located near any clinician known to prescribe MOUD. Approximately 80% of Indiana’s ZCTAs lacked waivered clinicians. Among waivered clinicians, fewer than 10% were willing to prescribe to adolescents. In practical terms, a parent seeking an MOUD clinician for an adolescent in Indiana would have to call an average of 11 health care clinicians listed on SAMSHA’s website before finding a clinician who might be willing to prescribe MOUD to individuals younger than 18 years. Even then, a parent would likely have to navigate other hurdles to obtaining MOUD for youth younger than 16 years, including the low likelihood of encountering a clinician close to their home.

### Community-Level Characteristics

Additional findings associated with community-level and clinician characteristics suggest potential points of intervention and future research directions. Results indicate significant associations between the areas in which clinicians practice and their willingness to prescribe MOUD to adolescents. Among all locations with a waivered clinician, clinicians practicing in less populated areas or more rural areas were more likely to prescribe to individuals younger than 18 years. Yet, the fact remains that Indiana’s most rural areas have no local access to an MOUD clinician at all (Table 2), a finding repeated throughout the literature.^23,30,31,32^ Clinicians practicing in less populated areas who are willing to treat a wider age range of patients with MOUD may be more aware of the paucity of addiction treatment in proportion to the relative need for treatment in their communities and, therefore, may feel a greater sense of responsibility to offer those services. Alternatively, clinicians practicing in lower-resourced areas might be more likely to prescribe to younger patients due to their proximity to other safety-net behavioral health services (eg, community health or federally qualified health centers [FQHCs]),^29^ which have been reported by clinicians as a requisite complement to effective MOUD treatment.

In contrast to previous research finding lower rates of OUD prescription among Black and Hispanic or Latine youth and lower income populations,^33^ the proportion of Black or African American residents where clinicians practice did not explain variation in clinician willingness to prescribe to adolescents. Similarly, we found no evidence of spillover effects of characteristics of neighboring communities on willingness to prescribe. Additional findings can be found in the eTable in Supplement 1.

### Clinician Characteristics

We also explored potential associations between clinician credentials and specialty training on their willingness to treat adolescents with MOUD. Results of both multivariable models indicated physicians were significantly more likely to prescribe to adolescents than APCs, despite efforts in the last decade to remove barriers to APC prescribing and increase workforce capacity to prescribe MOUD.^34^ We also found that clinicians with family medicine training were more likely to prescribe to adolescents than those who lacked training in family medicine, addiction medicine, or psychiatry. Our multivariable models indicated no significant difference in clinician willingness to prescribe MOUD to adolescents between family medicine clinicians and those with training in addiction medicine or psychiatry, further suggesting that family medicine clinicians offer a potential audience for interventions to increase access to MOUD for all ages. This finding aligned with continued calls for primary care clinicians to prescribe MOUD in clinic-based settings where patients were likely to receive routine care, especially in rural areas where access to specialty care is limited.^35^ Another benefit of family medicine clinicians, who were more numerous than behavioral health specialists, was their ability to care for adolescents into adulthood without a transition to a new clinician

We originally hypothesized that clinicians with training in pediatrics, which may suggest that they more regularly encounter adolescent patients, would be more likely to prescribe MOUDs to adolescents. However, only 13 of Indiana’s 832 waivered buprenorphine clinicians were trained or board certified in pediatrics, and none reported willingness to prescribe MOUD to adolescents. This finding highlights a need to incorporate addiction-related training into pediatric training programs to expand access to evidence-based, standard-of-care treatment for adolescents struggling with opioid use.^36,37^

### Limitations

This study has limitations. First, clinician self-report of their willingness to prescribe MOUD to adolescents does not equate to actual prescribing, and we did not have access to information regarding clinician prescribing rates or even how frequently clinicians encountered adolescent patients in need of MOUD. Second, our original sample of clinicians eligible for contact by the research team was derived from a publicly available national list, which may not be updated regularly; though this approach mimics the circumstances under which a member of the public (eg, a parent) might find an Indiana health care clinician to treat an adolescent with MOUD, we are unable to comment on the comprehensiveness of the clinician list. Similarly, since waiver requirements were recently lifted and website registration is voluntary, this study does not reflect potential changes in clinician attitudes and practice. Third, because the primary outcome is binary, it may not capture the nuance in clinician willingness to prescribe or the specific concerns that should be addressed to increase MOUD adoption for adolescents. Fourth, our exploration of community characteristics on clinician willingness to prescribe MOUD to adolescents included only areas in which a waivered buprenorphine clinician was located. In other words, we could only consider clinician willingness to prescribe MOUD to adolescents among those ostensibly willing to prescribe to adults. Though no local spillover effects were found for explanatory community-related variables, the final multivariable SLX model performed better than the standard logistic regression without spatially lagged variables. It seems likely that the model is capturing a global spillover effect not specific to a single explanatory variable, such that any spatial patterns observed are difficult to discern among the limited number of ZCTAs included in analyses. Future study designs should consider constructing a sample that allows more thorough exploration of community-level variables.

## Conclusions

These findings from Indiana show that adolescents continue to face major gaps in access to MOUD. As addiction medicine physicians and psychiatrists cannot meet the growing need for MOUD, primary care practitioners, especially those who already treat adults with OUD or have some comfort with adolescent patients (ie, family medicine clinicians) may be an ideal target of intervention.

## Notes

Supplement 1. **eTable.** Binary Logistic Regression Models

Supplement 2.
Data Sharing Statement
