# Supplementary material for: Clinician Willingness to Prescribe Medications for Opioid Use Disorder to Adolescents in Indiana
Source: JAMA Netw Open. 2024 Sep 25;7(9):e2435416. doi: 10.1001/jamanetworkopen.2024.35416 (PMC11425143; doi:10.1001/jamanetworkopen.2024.35416)
Supplement: Supplement 1. — eTable. Binary Logistic Regression Models [file jamanetwopen-e2435416-s001.pdf]

Supplemental Online Content

Aalsma MC, Bell LA, Schwartz K, et al. Clinician willingness to prescribe medications for opioid use disorder to adolescents in Indiana. *JAMA Netw Open*. 2024;7(9):e2435416.
doi:10.1001/jamanetworkopen.2024.35416

eTable. Binary Logistic Regression Models

This supplemental material has been provided by the authors to give readers additional information about their work.

© 2024 Aalsma MC et al. *JAMA Network Open.*

eTable. Binary Logistic Regression Models

		Standard Model		SLX Model	
		Estimate	Probability Chi-square	Estimate	Probability Chi-square
**Sociodemographic characteristics of clinician location and neighboring locations**					
Total population	Every 10,000 people increase	0.65 (0.50,0.86)	<0.001	0.68 (0.50,0.92)	0.01
Total population (W)[a]				0.71 (0.51,0.97)	0.03
% Black/AA	Every percentage increase	0.98 (0.94,1.02)	0.32	1.03 (0.97,1.10)	0.29
% Black/AA (W)				0.92 (0.87,0.99)	0.02
% in poverty	Every percentage increase	1.02 (0.96,1.07)	0.54	1.06 (1.00,1.13)	0.06
% in poverty (W)				0.91 (0.82,1.00)	0.05
Rurality[b]	Every 1 point increase (1-10)	1.22 (1.08,1.38)	0.002	1.25 (1.01,1.54)	0.04
Rurality (W)				0.97 (0.74,1.27)	0.80
**Clinician characteristics**					
Clinician credentials (MD/DO referent)	APC[c]	0.82 (0.55,1.20)	0.31		
Clinician training (Family Medicine referent)	Addiction Medicine or Psychiatry	1.12 (0.70,1.79)	0.63		
	Other[d]	0.45 (0.25,0.81)	0.008		

[a] W = spatially-lagged variable, generated through by spatial weight matrices
[b] Rurality is described with Rural Urban Commuting Area, version 2 (RUCA2) codes, an index from 1-10 in which higher scores indicate a location is more rural[32]
[c] Advanced Practice Clinician (i.e., nurse practitioner, physician's assistant)
[d] Other = Clinician had training in any category other than addiction medicine, psychiatry, or family medicine

© 2024 Aalsma MC et al. *JAMA Network Open.*
