# Supplementary material for: Clinician Willingness to Prescribe Medications for Opioid Use Disorder to Adolescents in Indiana
Source: JAMA Netw Open. 2024 Sep 25;7(9):e2435416. doi: 10.1001/jamanetworkopen.2024.35416 (PMC11425143; doi:10.1001/jamanetworkopen.2024.35416)
Supplement: Supplement 2. — Data Sharing Statement [file jamanetwopen-e2435416-s002.pdf]

Data Sharing Statement

Data
Data available: Yes
Data types: Deidentified participant data, Data (not involving human participants)
How to access data: maalsma@iu.edu
When available: With publication

Supporting Documents
Document types: Statistical/analytic code
How to access documents: maalsma@iu.edu
When available: With publication

Additional Information
Who can access the data: researchers whose use of data is approved
Types of analyses: for validation purposes
Mechanisms of data availability: signed data access agreement
